# Which Biological Properties of Heart Valves Are Relevant to Tissue Engineering?

**DOI:** 10.3389/fcvm.2020.00063

**Published:** 2020-04-21

**Authors:** Adrian H. Chester, K. Jane Grande-Allen

**Affiliations:** ^1^Heart Science Centre, The Magdi Yacoub Institute, Harefield, United Kingdom; ^2^Department of Bioengineering, Rice University, Houston, TX, United States

**Keywords:** engineered tissue heart valves, scaffolds, valve interstitial cells, valve endothelial cells, extracellular matrix, calcification, mechanobiology

## Abstract

Over the last 20 years, the designs of tissue engineered heart valves have evolved considerably. An initial focus on replicating the mechanical and structural features of semilunar valves has expanded to endeavors to mimic the biological behavior of heart valve cells as well. Studies on the biology of heart valves have shown that the function and durability of native valves is underpinned by complex interactions between the valve cells, the extracellular matrix, and the mechanical environment in which heart valves function. The ability of valve interstitial cells to synthesize extracellular matrix proteins and remodeling enzymes and the protective mediators released by endothelial cells are key factors in the homeostasis of valve function. The extracellular matrix provides the mechanical strength and flexibility required for the valve to function, as well as communicating with the cells that are bound within. There are a number of regulatory mechanisms that influence valve function, which include neuronal mechanisms and the tight regulation of growth and angiogenic factors. Together, studies into valve biology have provided a blueprint for what a tissue engineered valve would need to be capable of, in order to truly match the function of the native valve. This review addresses the biological functions of heart valve cells, in addition to the influence of the cells' environment on this behavior and examines how well these functions are addressed within the current strategies for tissue engineering heart valves *in vitro, in vivo*, and *in situ*.

## Introduction

The quest to tissue engineer heart valves is stimulated by the limitations of currently available valve substitutes, which due to a lack of viable cells, either fail to replicate the sophisticated function of the native valve or undergo degeneration and eventual failure. The aim of heart valve tissue engineering projects is to produce a valve that can mimic more accurately the complex biological function of the native valves (1, 2). The ultimate aim is to produce a valve that has sustained durability, is haemocompatible, non-immunogenic, has the ability to grow, and is resistant to calcification. To date, only nature has produced such a valve, through a tightly controlled developmental process, that is able to function in the majority of individuals throughout their life without any significant problems. Unsurprisingly, the advent of heart valve tissue engineering has coincided with investigations into the biological properties of heart valves in a quest to understand how nature has achieved the development of what tissue engineers may regard as the “Holy Grail.”

The early studies on the biology of heart valve cells set out to establish how the cellular and structural components of valves influence the durability of the valve and its structure-function relationship. They were also motivated by a need to understand the properties of valve interstitial cells and the extracellular matrix with a view to finding appropriate cells and scaffold materials that would be able to recapitulate the function of native structure in tissue engineered valves. The initial focus for tissue engineering heart valves sought to mimic the structure and mechanical function of the valve cusps, i.e., functional tissue engineering. A growing number of studies focusing on biology and mechanobiology, however, have characterized heart valves at the molecular, protein, cellular, and tissue level (3, 4). As a result, we are now beginning to understand in greater depth the biological mechanisms that are essential to the successful function and durability of heart valves. These studies have highlighted the key roles played by the cells and the importance of having a living valve, a concept supported by the clinical experience with the Ross procedure (5–7).

This article will review the current understanding of the mechanisms that influence valve function, with respect to the structural and cellular components of the valve and the interaction between the cells and the extracellular matrix, in an attempt to identify which of these factors are most relevant to the development of tissue engineered heart valves. Since the focus of most tissue engineering projects is to produce a valve capable of implantation into either the aortic or pulmonary position, this article will focus on the biological mechanisms relevant to semi-lunar valves.

## Heart Valve Structure-Function Relationship

**Figure 1:**
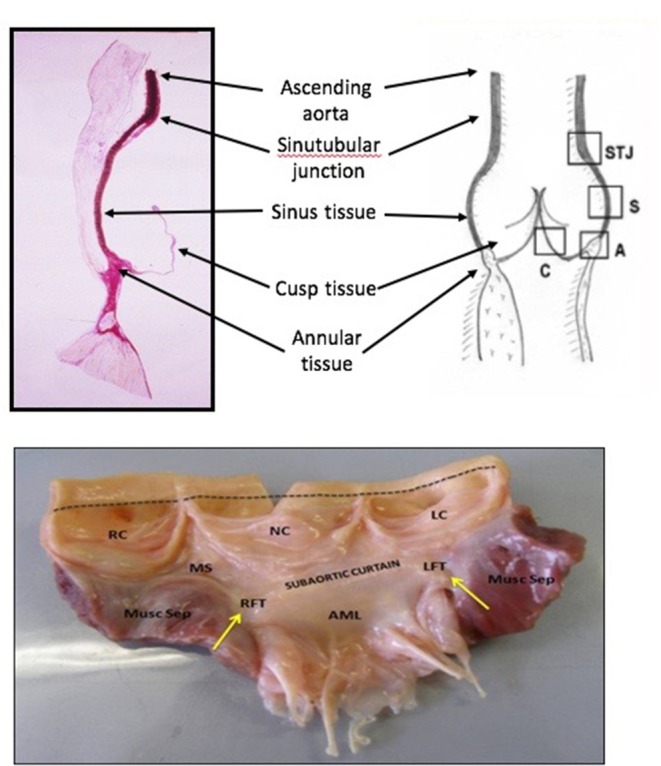
Histological, schematic and photographic representations of the aortic root. AML, anterior mitral leaflet; LC, left coronary cusp; LFT, left fibrous trigone; MS, membranous septum; Musc Sep, muscular septum; NC, non-coronary cusp; RC, right coronary cusp; RFT, right fibrous trigone.

The aortic valve consists of a number of distinct structural components. The whole valve machinery is termed the “aortic root” and is located between the left ventricle and the ascending aorta. The valve cusps are attached to a crown-shaped annulus, with the highest point of the attachment (known as commissures) marking the boundary of the valve and the ascending aorta. This is identified as a small ridge termed the sinutubular junction. In the wall of the root, above the point of attachment of each cusp, are three bulges called the sinuses of Valsalva. Two of the sinuses give rise to left and right coronary arteries (Figure 1).

**Figure 2:**
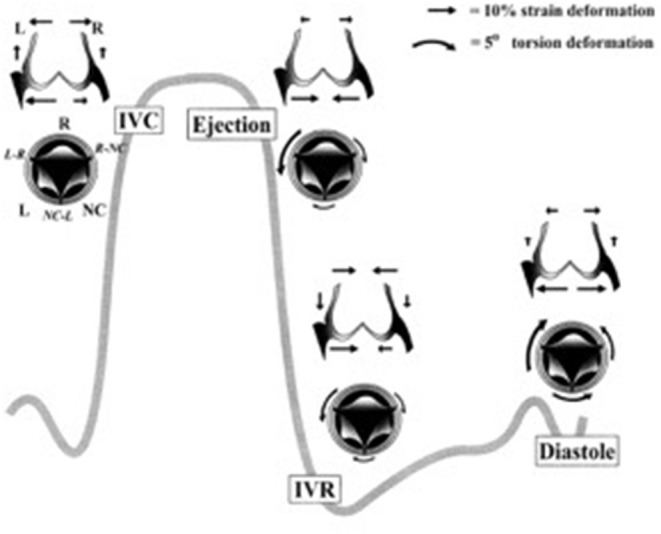
Aortic annular deformation of left (L), right (R), and non-coronary (NC) sectors of aortic annulus through different stages of the cardiac cycle indicating expansion of the annular region of the root during diastole to accommodate blood prior to ejection from the left ventricle (9). IVC, isovolumic contraction; IVR, isovolumic relaxation.

The dynamic function of the aortic valve relies on the co-ordinated movement of these different components. From the subvalvular regions (left ventricular outflow tract, subaortic curtain & fibrous trigones) up to the supravalvular regions (sinus of Valsalva and the sinutubular junction), the geometry of each part of the valve determines how it moves in response to the flow of blood during the cardiac cycle (8, 9). For example, the shape of the sinuses determines the formation of the vortices that are important for valve closure and maintaining coronary flow. In addition, certain structures in each section of the valve (cusp, annulus, sinus, sinutubular junction) change their size, shape, and stiffness during specific portions of the cardiac cycle to guarantee optimal valve opening, ejection of blood from the left ventricle, rapid valve closure, coaptation of the leaflets, and adequate coronary perfusion. Before the aortic valve opens, the aortic root prepares by accommodating a large volume of blood exiting from the left ventricle thereby improving transvalvular hemodynamics and reducing turbulent damage to the aortic cusps. This is achieved by asymmetric changes in the shape of the root. For instance, during the early isovolumic contraction phase, circumferential expansion of the base is greatest in the left annular region, and least in the non-coronary annular region (Figure 2). These changes are accompanied by an increase in circumferential diameter at the level of the commissures. This conformational change in diameter is proportional to the end-diastolic volume (9, 10).

Superimposed on the movements of the valve apparatus are the regulatory influences that comprise active dynamism. Central to the phenomena of active dynamism is the fact that the valve is a “living” structure that contains a number of key “players” that orchestrate the response of the valve to its mechanical environment, allowing it to respond immediately (such as by release of nitric oxide), or to adapt to chronic changes to the environment in which it resides, as described below.

## Functional Roles of Valve Cells

**Figure 3:**
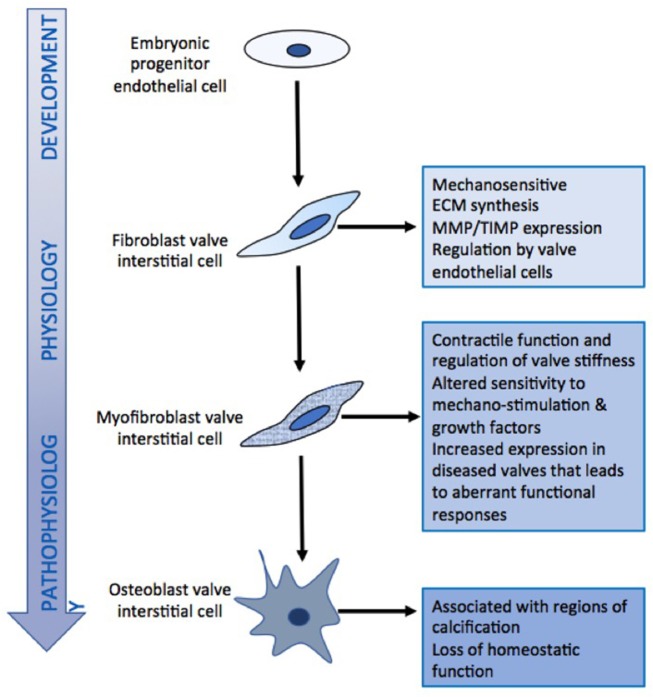
Schematic diagram of the relationship between different valve cell phenotypes and their functional characteristics.

**Figure 4:**
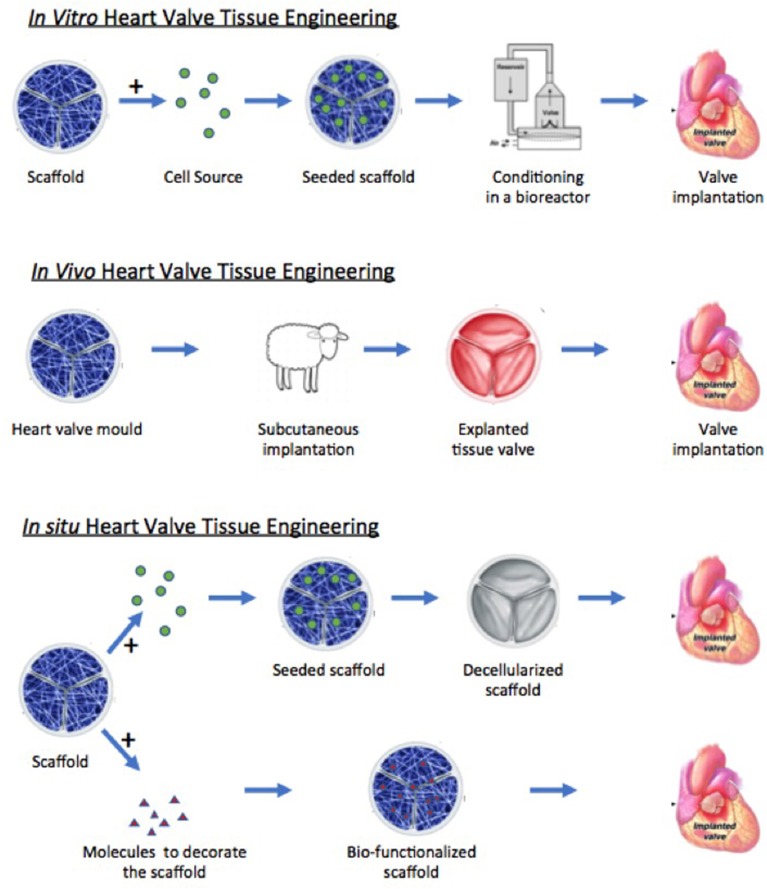
Schematic diagram illustrating the different approaches of tissue engineering. Modified from Jana et al. (20).

Little more than 20 years ago, valve cusps were generally considered to be collagenous flaps of tissue that passively responded to the flow of blood through the heart. With the expansion of interest in heart valve biology, it is now accepted that the interstitial cells that reside within valve cusps and the endothelial cells that cover the surface of the cusps play a key role in maintaining the integrity and durability of valve cusps (11). Valve cells can be categorized into a number of distinct phenotypes (Table 1), whose characteristics and function have been reviewed elsewhere (3, 4). From a functional point of view, there are two distinct populations of interstitial cells within the cusps: a fibroblast-like cell, which is the predominant cell phenotype in mature healthy valves, and a myofibroblast phenotype that is associated with developing valves and those developing pathological changes (Figure 3) (4, 12). Valve interstitial cells normally express the same contractile proteins and transcription factors found in skeletal muscle (13). Collectively, the population of interstitial cells within valve cusps confers the valve with two key functional properties: the ability to contract and the capacity to synthesize extracellular matrix components (14–17). It has been shown that the contractile function of valve cusps is capable of regulating the mechanical properties of cusp tissue, in that increased contractile function causes the stiffness of the tissue to increase (18). This finding had led to the hypothesis that cusp stiffness is dynamically regulated by bioactive molecules that serve to optimize valve function. The ability of valve interstitial cells to secrete extracellular matrix proteins as well as matrix crosslinking and remodeling enzymes indicates the role of these cells in maintaining the integrity of the extracellular matrix and the durability of cusp tissue. The secretory properties of valve interstitial cells are regulated by growth factors such as TGFβs and mechano-transduction pathways activated by the cyclical stretching of the cusps during the cardiac cycle (Figure 4). For example, mechanical stretch can stimulate the production of collagen, secretion of the matrix remodeling enzymes MMPs and TIMPs, and the production of growth factors (15, 16, 19).

**Table 1:** Phenotypes and characteristics of valve cells.

**Cell type**	**Location**	**Function**
Embryonic progenitor endothelial/ mesenchymal cells	Embryonic cardiac cushions	Give rise to VICs through an activated stage or EMT
Progenitor-VIC	Bone marrow, blood, valve leaflet	Provide fibroblast-VICs for valve repair. May be CD34^−^, CD133^−^, and/or S100^+^
Fibroblast-VIC	Valve leaflet	Maintain valve structure and function and prevent angiogenesis
Myofibroblast-VIC	Valve leaflet	SMA-containing VICs with activated cellular repair processes. Respond to valve injury and abnormal hemodynamic or mechanical forces
Osteoblast-VIC	Valve leaflet	Calcification, chondrogenesis and osteogenesis in the leaflet. Secrete ALP, osteocalcin, osteopontin and bone sialoprotein
Valve endothelial Cells	Surfaces of valve leaflet	Blood/valve interface, source of biological factors. Prevents cell adhesion, platelet activation, protects against calcification

*VIC, valvular interstitial cells; qVIC, quiescent valvular interstitial cells; VEC, valvular endothelial cells; EMT, endothelial to mesenchymal transformation; ALP, alkaline phosphatase; SMA, smooth muscle α-actin*.

In an identical manner to the vascular endothelial cells, those that cover the surfaces of the valve cusps serve to act as barrier between the blood components and the underlying microstructural elements and cells of the valve cusps (21). Valve endothelial cells share the same phenotypic markers as other endothelial cells but reside in a vastly different hemodynamic environment. Several studies have shown that valvular endothelium possesses unique properties that distinguish the cells on one side of the valve from the other, and from other endothelial beds, particularly the endothelium lining the aorta with which it lies in direct continuity, which may reflect organ-specific endothelial cell differentiation and function (21–23). These differences may be due to the patterns of flow experienced by each side of the valve, but also to differences in the mechanical properties of the cells on the aortic and ventricular surfaces of the valve. The cells that reside on the ventricular surface are significantly stiffer than those on the opposing side. The stiffness of the cells was related to the expression of cytoskeletal proteins, that could not be modulated by changing the pattern of flow to that experienced by the opposing side in a bioreactor, inferring that the difference in cell stiffness is a property of the cells, rather than an adaptation to the flow environment of the cells (24). Given the heterogeneous composition of the valve layered structure, the stiffnesses of the extracellular matrix will vary on different sides of the leaflet, which could also affect the behavior of the endothelial cells. Like their vascular counterparts, valve endothelial cells synthesize thrombotic and anti-thrombotic proteins to maintain hemostasis. This aspect of valve endothelial cell function was shown to be sensitive to the stiffness of the adhesive substrate on which the cells were grown; the cells cultured on softer hydrogel platforms had significantly higher gene expression for a wide range of anti-thrombotic and thrombotic proteins than the cells cultured on stiffer hydrogels and tissue culture polystyrene controls (25). Valve endothelial cells also release bioactive molecules in response to the flow of blood over their surfaces and to stimulation by circulating bioactive molecules. Differences in flow patterns between both sides of the valve are sensed by the luminal surface of endothelial cells, which activates signaling pathways to stimulate the synthesis of nitric oxide via the action of the enzyme nitric oxide synthase (26–29). A co-culture model of aortic valve interstitial and endothelial cells was used to demonstrate that the release of nitric oxide by endothelial cells will suppress the interstitial cells from expressing the myofibroblast phenotype (30). In addition to nitric oxide, other bioactive molecules such as prostacyclin and endothelin-1 are expressed and released by flow-independent mechanisms (31–33). These substances are able to interact with both the blood and the underlying valve interstitial cells and have a range of differing effects on the valve.

## Diverse Functionality of The Extracellular Matrix

The extracellular matrix is responsible for the mechanical strength of the valve cusp. There is a precise arrangement and composition of the extracellular matrix proteins, which gives the cusps three distinct superposed layers: the fibrosa, spongiosa, and ventricularis (5). The fibrosa is on the aortic side of the cusp while the ventricularis is on the ventricular-facing side. The spongiosa layer lies between these two layers. The fibrosa is comprised principally of circumferentially-oriented collagen fibers. These collagen fibers are crimped, meaning that deformation will cause them to straighten out before they actually bear load. Once the collagen fibers are uncrimped, they provide much of structural strength to the cusps. The fibrosa also has a corrugated structure in which the folds are able to flatten out when the valve cusp undergoes radial stretch. In contrast, the ventricularis is mainly composed of radially-aligned elastic fibers, conferring elasticity to the leaflets. Taken together, the folded nature of the fibrosa, the collagen fiber crimp, and the elastic fiber composition of the ventricularis bestow a substantial degree of extensibility to the cusps, especially in the radial direction. The middle spongiosa layer is rich in proteoglycans and glycosaminoglycans, which have a high hydrous content that allows smooth sliding of the fibrosa and ventricularis during the various phases of the cardiac cycle, thereby minimizing repeated microtrauma related to valve deformation (34).

In addition to the physical strength that the extracellular matrix gives to the valve cusps, it also provides the framework to which the cellular components of the valve attach. This is achieved through the binding of cell-surface proteins, such as integrins, to specific peptide sequences on extracellular matrix proteins. It has been shown that valve interstitial cells express α_1_, α_2_, α_3_, α_4_, and α_5_ integrins to varying degrees and predominantly β_1_ integrins but not β_3_ or β_4_ integrins (35). These integrin-matrix connections not only provide anchoring points for cell to connect to the extracellular matrix, they also serve to stimulate the valve cells. It has been shown that integrins regulate proliferation, differentiation, and action of growth factors in other non-valve cell types (36–38). This observation has led to the concept that the extracellular matrix is able to instruct the cells that populate the valve via communication through integrins to intracellular signaling pathways (35, 39). These cell-matrix connections also perform a key role in the transduction of mechanical signals to the cells. The valve experiences a number of different forces during each cardiac cycle, which exposes the interstitial cells to combinations of stretch and compression (during valve closure) and exposes the endothelial cells to either shear stress (the effect of either laminar or oscillatory blood flow over the ventricular and aortic surfaces of the valve leaflets, respectively) (40, 41). These mechanical signals are transferred from the external environment to the cells through extracellular matrix connections to the integrins, which connect to the intracellular cytoskeleton via a complex of proteins within focal adhesions. As mentioned above, in response to mechanical stimulation, valve interstitial cells secrete collagen and increase their expression of matrix remodeling enzymes in response to the signals experienced by the valve and transduced to the cells (14, 15). Due to the shielding effect of the extracellular matrix on the cells, only a portion of tissue strain experienced by the valve during diastole is experienced by the cells within the matrix (42). Valve endothelial cells also adhere to extracellular matrix through binding of integrins as well as syndecans and potentially additional cell-matrix adhesion proteins (25).

This relationship between the extracellular matrix and the valve cells is fundamental to the longevity of the valve and its normal myriad of functional roles. The valve interstitial cells provide a source of matrix proteins and remodeling enzymes that help to sustain the integrity of the entire matrix during the lifetime of the valve. Similarly, adhesion of the valve endothelial cells to the basement membrane laminins promotes the formation of a continuous endothelium that mediates hemostasis and inflammation. These biological cues are important during valve development and continue throughout the life of the tissue, maintaining specialized cellular function, and tissue regeneration. To date there have been a limited number of studies on the expression and functional role of integrins and other cell-matrix adhesive proteins in valve cells (30, 35, 43–45).

## The Impact of Innervation and Vascularization on Heart Valve Function

Heart valves also contain nerves and blood vessels. Immunohistochemical studies have shown a rich innervation of cusp tissue with distinct, age-dependent patterns of innervation by both primary sensory, and autonomic components in the aortic valve (46). Isolated porcine aortic valve cusps have been shown to have sympathetic and parasympathetic contractile responses mediated by neuronal stimulation. In addition, nitric oxide-containing nerves are also present, which mediate relaxation of valve cusps (47). In the mitral valve, stimulation of the vagus nerve was reported to alter leaflet stiffness *in vivo* in sheep (48). The intrinsic and heterogeneous networks of afferent and efferent nerves are likely responsible in part for the control of valve stiffness and adaptation to changing hemodynamic conditions (49).

In addition, the presence of cells throughout the mm-thick leaflet suggests that valve cells have metabolic activity and therefore require a supply of O_2_ above a level that can be supplied by simple diffusion, i.e., it must be delivered through cyclic loading-driven convection and a vascular network. Indeed, convection due to interstitial fluid flow was calculated to enhance the oxygen transport within the leaflets by up to 68% (50). Additional studies have shown that the thickest regions of the valve would be hypoxic without an additional supply of O_2_ delivered via a vascular network (51–53). The relationship between vessel density and thickness of the leaflet has found that vessels are present when the cusp leaflet exceeded 0.5 mm (54). The requirement for O_2_ may be important for cell function to maintain the normal structure and function of the valve. Pathological valves conditions such as calcific and rheumatic valve disease are both associated with angiogenesis (20, 55), due possibly to hypoxic conditions within thickened valve cusps and the expression of hypoxia induced factor 1α (HIF-1α) (56), or the loss of anti-angiogenic factors such as chrondromodulin-1 (57).

## Relevance to Heart Valve Tissue Engineering

The structure-function relationship, valve cells, and the extracellular matrix are all fundamental aspects of normal heart valve function. The relative influence of these factors in projects focused on the tissue engineering of heart valves, however, depends in part upon the approach being taken and the type of valve that is going to be produced. Broadly speaking, three approaches currently exist: *in vitro, in vivo*, and *in situ* tissue engineering (Figure 4). With respect to the valves that are being made, these can be then be divided into stented and free-standing valves. However, irrespective of the approach taken, the goal remains the same: to produce a valve that replicates the mechanical and biological function of the native valve and that will be durable and will be able to grow with the patient.

## *In vitro* Tissue Engineering

The *in vitro* approach relies on finding a suitable cell source and a scaffold onto which to seed the cells, prior to conditioning in a bioreactor and subsequent implantation into the patient (58). The choice of the cells and scaffold needs careful selection. From our knowledge of heart valve biology, we have some information regarding the expectations of the cells to be used in this approach. Alongside considerations relating to availability of cells, their immunogenicity, and their ability to proliferate in culture, the functional properties of cells must also be assessed. Previous studies have advocated for a range of candidate cell populations to replicate the role of valve interstitial cells, such as bone marrow-derived mesenchymal stem cells, adipose-derived stem cells, umbilical vein progenitor cells, and saphenous vein smooth muscle myofibroblasts (59–64). For cells that need ultimately to function as valve interstitial cells, it is particularly relevant to have the ability to populate the scaffold material, secrete extracellular matrix proteins in response to either growth factors or mechanical stimulation, and express matrix remodeling enzymes in the same magnitudes as valvular interstitial cells. This property will allow these cells to act in a similar way to valve interstitial cells in remodeling and maintaining the extracellular matrix of the new valve.

The *in vitro* approach also requires an endothelial cell source to cover the surfaces of the tissue engineered valve. Endothelial progenitor cells have been investigated for this purpose (65, 66), but there has been no comprehensive assessment as to how these cells respond to the patterns of flow that are experienced by each side of the valve. Since side-specific valve endothelial cells demonstrate specific mechanical and functional characteristics (24), it will be important to assess the behavior of endothelial cell sources under these distinct conditions. The relevant functional responses of valve endothelium relate to their ability to suppress calcification responses in interstitial cells and their ability to regulate extracellular matrix secretion (11).

**Figure 5:**
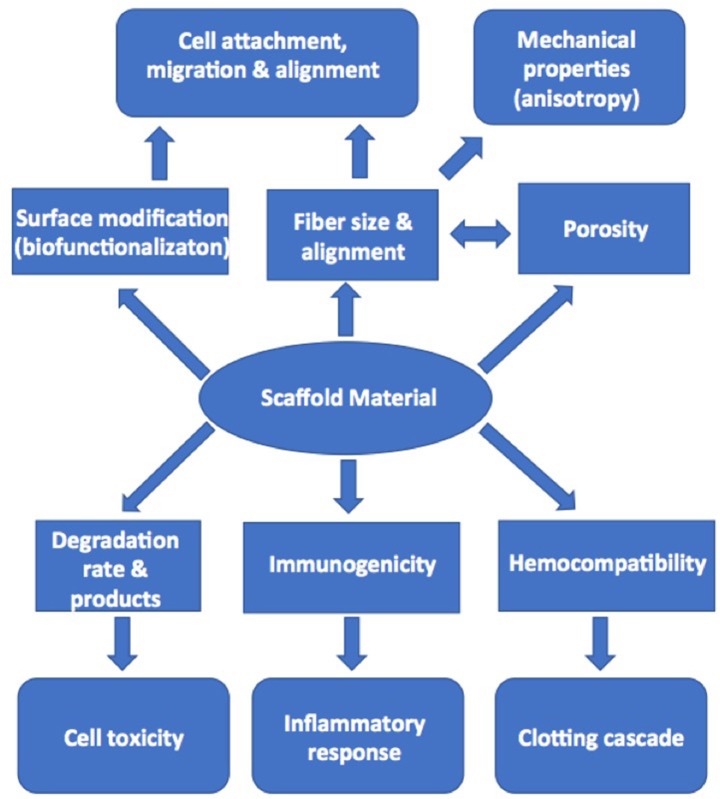
Diagram illustrating the factors in scaffold production that can influence cell adhesion, migration, and activation in tissue engineering.

Equally important in this approach to tissue engineering is the choice of the scaffold material onto which the cells are seeded. Although scaffolds need to have the necessary mechanical strength and distensibility, there are a number of considerations with the choice and design of scaffold material that will impact the compatibility with cells (Figure 5). Recapitulation of the known anisotropic profile of cusp tissue is also essential and will allow tissue engineered valves to expand more in the radial direction than circumferentially, which will assist with the coaptation of the valve cusps during valve closure. Several methods, including newer approaches such as melt electrowriting, have been used to produce anisotropic scaffolds that either mimic, or structurally replicate collagen-like fibrous microarchitecture and associated material behavior (67–72). Some projects have developed biological scaffolds from different preparations of collagen (73–75). These scaffolds have a potential advantage in that the cells will recognize the integrin-binding sequences on the collagen and bind to them accordingly. However, some collagen scaffolds have not been found to have the sufficient intrinsic strength to make their use a viable proposition. This weakness can be potentially overcome by cross-linking the collagen or incorporating additional extracellular components and thereby producing a biological scaffold of sufficient strength (76). The alternative, and more common approach, is to use synthetic polymer materials. Although these synthetic materials are typically stronger than biological scaffolds, especially when they are prepared as fibrous meshes, they lack the binding sites needed by the cell to accept biological cues as they would through binding to extracellular matrix proteins. Attempts to overcome this absence of biological cues have been made by “decorating” the scaffold with peptide recognition sequences or with extracellular matrix proteins (77).

**Figure 6:**
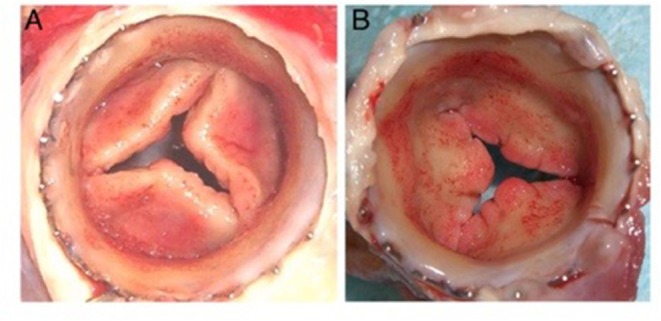
Distal **(A)** and proximal **(B)** views of cusp thickening and retraction after 8 weeks transapical implantation into the pulmonary position in a sheep (78).

The bioreactor is an additional integral partner in the *in vitro* approach to developing a tissue engineered heart valve. The general dogma is the use of a bioreactor to apply mechanical loading to the engineered valve (either physiological or sub-physiological), since that mechanical stimulation has been shown to increase cell ingrowth in to the scaffold material and to increase the deposition of cell-synthetized extracellular matrix within the scaffold, resulting in mechanical properties that more closely approximate those of native heart valves (78). Up until several years ago, bioreactor-conditioned tissue engineered heart valves were then implanted into animal models as part of the preclinical development stage of these devices (79, 80). Multiple research groups, however, reported that the cells within the valves became strongly activated, resulting in leaflet retraction after the engineered tissues were implanted in animals, and that the extracellular matrix was typically lacking in elastin (Figure 6) (78, 79, 81–84). Based on that experience, some of these groups have refined their approach and decellularize their bioreactor-grown engineered materials prior to implantation in the preclinical animal model, with the goal of *in situ* recellularization by the host cells (85).

For the reasons above, the processes involved with *in vitro* tissue engineering are recognized to be time consuming, making it unlikely that an off-the-shelf, commercially viable product will be produced. This acknowledgment has ultimately led to the development of *in vivo* and *in situ* tissue engineering strategies that do not require an external cell source.

## *In vivo* Tissue Engineering

The *in vivo* tissue engineering approach uses the body's ability to encapsulate foreign material and utilize fibroblasts to produce extracellular matrix proteins. For this strategy, a valve-shaped mold is implanted subcutaneously, eventually resulting in coverage of the mold with a membranous tissue that can then be harvested and subsequently used as a replacement valve (86–88). While this approach appears attractive from a perspective of convenience and the population of the material with host cells, other than mimicking the geometry of the valve with the shape of the mold, little else relies on information gained from knowledge of the biology of heart valves. There is no control over the cells that are present in the tissue that is formed, nor over the composition of the resulting extracellular matrix, or its mechanical properties.

## *In situ* Tissue Engineering

Unlike the method described above, the *in situ* approach adopts a cell free method, with the implantation of valves made from un-seeded scaffolds and then relying on the body's ability to recruit cells to populate and remodel the scaffold and ultimately generate optimally functional tissue (85, 89, 90). Thus, the key element in this approach is the scaffold material and its ability to attract and bind cells of the required phenotype from the circulation after implantation. The scaffold materials for *in situ* tissue engineering may either be newly fabricated from material components (native or synthetic polymers) or decellularized from native or bioreactor-grown valves. Understanding the function and composition of the extracellular matrix and how it instructs cells is of key importance to this strategy with either type of scaffold. For synthetic polymers, biofunctionalization—incorporating peptides, matrix proteins, or recognition sequences—is one method to confer biological properties to scaffold material such that it is able to mimic natural matrix proteins (91, 92). An alternative strategy is to use the hybrid approach described above whereby cells are seeded onto the scaffold material and stimulated to secrete extracellular matrix proteins using a bioreactor (89). The cells are then removed using a decellularization protocol, leaving a polymer matrix with a coating of matrix proteins. In this method, the choice of cells initially employed for this purpose may be an important consideration since having a similar extracellular matrix production profile as valve interstitial cells may be an advantage.

Given that *in situ* tissue engineering does not involve the implantation of a cell-seeded device, this approach is suitable for the production of an off-the-shelf product and presents a more attractive economic proposition for the commercial development of a tissue engineered valve. At the current time, the *in situ* approach to tissue engineering heart valves has made the most progress with a number of *in vivo* studies in animals, and with delivery of the valve using transcatheter implantation, showing encouraging results (89, 93–95). This approach has also been the avenue for the small number of past and present clinicial trials of tissue engineered heart valves, most often employing decellularized homografts for use in the pulmonary valve position (96).

## Importance of Valve Shape and Design

With the knowledge of how the different structures within the aortic root contribute to the function of the valve cusp, some tissue engineered valve designs aim to incorporate sinuses, a sinutubular junction and attachment of the valve cusps to the wall of the structure. The movements of these structures throughout the cardiac cycle in the native valve are governed in part by biological mechanisms, such as the contractile properties of valve cells and their regulation by nerves, but also by the differences in the structural composition and mechanical properties of the component parts. How well a tissue engineered valve replicates these aspects of native valves depends on which aspect is being considered. It would be unrealistic to expect that the *in vivo* or *in situ* approaches result in replicating the precise biological properties of the cells, nerves or vascular supply. However, the supply of O_2_ to meet the metabolic requirements of the cells that populate tissue engineered valves could be an important factor in maintaining viable cells in these valves, especially in pediatric patients in whom the ability of the valve to grow will be important. Cells that populate the valves will either need to be able to function under low O_2_ levels or respond to hypoxia by expression of HIF-1α, which can stimulate angiogenesis via the induction of VEGF. A similar angiogenic response occurs when valve cusps become thickened during the calcification process or in rheumatic valve disease (56, 97, 98).

The production of scaffold material for valve tissues, however, should be able to replicate the regional difference in mechanical properties to reflect those seen in different components of the aortic valve and aortic root. This mimicry has been achieved for the production of anisotropic material for valve cusps via the production of highly aligned fibers in polymer scaffolds (67–71, 99).

An alternative design to a free-standing aortic root is to produce a stented valve in which the tissue engineered valve cusps are attached to an annular-shaped stent and sewing ring. While this design potentially negates consideration of the co-ordinated movements of the aortic root structures or the ability of the valve to grow in younger patients, this approach has been successfully used for a number of bio-prosthetic valve designs with cusps derived from animal tissue (100). If the cusp material in tissue engineered stented valves does not experience the degenerative changes associated with the glutaraldehyde-fixed animal tissues used in currently available bio-prosthetic valves, then this approach may well-prove successful in patients where the growth of the valve is not a consideration.

## Prevention of Valve Calcification

Investigation into the biology of heart valves have highlighted the complexity of valve cell function under physiological and pathophysiological conditions. These studies have shown, for example, that valve endothelial cells participate in protecting the valve against mediators that can initiate the calcification process (100). Given that tissue engineered valves will face the same physiological environment as native valves, including the diverse risk factors that can lead to calcific aortic stenosis, it will be important to design tissue engineered valves to include mechanisms for preventing calcification and other valve diseases. Such design strategies could include the seeding of endothelial cells onto the surface of scaffolds, the attraction of circulating endothelial progenitor cells onto implanted valves, incorporation of anti-calcification agents into the scaffold material, or the development of pharmacological agents that can be given to the patient to prevent calcification. The method adopted will depend of the approach taken to make the tissue engineered valve (*in vitro* compared to *in vivo* or *in situ* tissue engineering) and the identification and development of efficacious anti-calcification agents.

## Designing for Valve Durability

At the current time a number of hurdles remain before tissue engineered valves are able to be considered as truly biological heart valves that are able to match the durability and function of the native valve. These principally relate to the control of the cells that populate the valve scaffold. Irrespective of which approach is adopted, implanting of the valve scaffold into the circulation will elicit a reaction and initial population of the scaffold material by inflammatory cells. Regulating this response, so that the new valve is populated with cells capable of mimicking the function of valvular interstitial cells, remains a significant challenge. Without the ability of cells to provide the same homeostatic and regulatory role provided by the interstitial and endothelial cells in native valves, the long-term durability and function of tissue engineered heart valves may be compromised.

## Conclusions

The quest to tissue engineer a living heart valve that possesses the same functionality and durability of the native heart valve has given rise to a number of alternative approaches and valve designs, which are now beginning to attempt to bridge the gap between laboratory projects and clinical trials—a major step in completion of these projects (101, 102). In doing so, each of these potentially new valves carries certain characteristics and functional properties of the native valve. These characteristics include the ability of cells to secrete extracellular matrix proteins, the capability of scaffold material to recruit and instruct circulating cells, and the replication of the mechanical properties of cusp material and the associated structures of the aortic root. However, incorporation of all the biological properties of native valves is not realistically achievable within the confines of the laboratory. In reality, achieving a successful outcome for tissue engineering projects such as heart valves will require an integrated approach relying on a combination of the different strategies that currently exist. Sustained partnerships between clinical, translational, and basic science faculty as well as industry partners will be needed to infuse the clinically evaluated tissue engineered heart valves with the newer aspects of biological functionality and material-based control of cell behavior that have just been reported in the last few years. In the future, an expanded set of data from clinical trials will be tremendously insightful into our understanding of the healing, cell metabolism, and growth within tissue engineered valves *in vivo*. Only time and clinical experience will tell which approach has incorporated the key functions of the native valve, allowing patients to receive a replacement valve that gives them a normal life expectancy, free from the need for life-long anticoagulation therapy or re-operation.

## Author Contributions

AC and KG-A both wrote, revised, and critically appraised the manuscript.

## Conflict of Interest

The authors declare that the research was conducted in the absence of any commercial or financial relationships that could be construed as a potential conflict of interest.
